# Veterans Health Administration Service Use and Suicide Risk After Military Separation

**DOI:** 10.1001/jamanetworkopen.2026.42356

**Published:** 2026-11-05

**Authors:** Lisa A. Brenner, Jeri E. Forster, Claire A. Hoffmire, Jaimie L. Gradus, Trisha A. Hostetter, Mary Jo Larson, Alexandra L. Schneider, Alexandra A. Smith, Colin G. Walsh, Rachel Sayko Adams

**Affiliations:** 1 University of Colorado Anschutz Medical Campus, Departments of Physical Medicine and Rehabilitation, Psychiatry, and Neurology, Aurora; 2 VA Brain Health Coordinating Center, Department of Veterans Affairs, Aurora, Colorado; 3 VA Rocky Mountain Mental Illness Research, Education and Clinical Center, Aurora, Colorado; 4 Department of Physical Medicine and Rehabilitation, University of Colorado Anschutz Medical Campus, Aurora; 5 Department of Epidemiology, Boston University School of Public Health, Boston, Massachusetts; 6 Institute for Behavioral Health, Heller School for Social Policy and Management, Brandeis University, Waltham, Massachusetts; 7 Departments of Biomedical Informatics, Medicine, and Psychiatry, Vanderbilt University Medical Center, Nashville, Tennessee; 8 Department of Health Law, Policy, and Management, Boston University School of Public Health, Boston, Massachusetts

## Abstract

This cohort study investigates Veterans Health Administration suicide prevention resources for US Army veterans and potential differences among those who did or did not seek care.

## Introduction

Substantial resources have been deployed to enhance suicide prevention–related care within the Veterans Health Administration (VHA).^1^ Many of these strategies have not been implemented in other health care settings.^2^ To increase understanding regarding potential differences in suicide among a broad cohort of Army veterans who did or did not seek VHA care following separation from military service, we examined (1) age-adjusted average annual suicide rates by VHA use group; and (2) time-dependent hazard rates and trends for suicide after separation by VHA use group (any VHA or fee for service [ie, outside care paid for by VA] vs no VHA utilization), as well as by timing and type of VHA utilization (any VHA outpatient care [no fee] and any VHA inpatient care [no fee]).

## Methods

In this cohort study, data were from the Substance Use and Psychological Injury Combat Study, a longitudinal cohort of all Army soldiers who returned from an Afghanistan or Iraq deployment in fiscal years 2008 through 2014 and separated from service by September 2017 (N = 649 811).^3^ Suicide deaths were identified through the National Death Index via the Departments of Veterans Affairs and Defense Mortality Data Repository (*International Statistical Classification of Diseases and Related Health Problems, Tenth Revision *[*ICD-10*] codes X60-X84 and Y87.0).^4^ Follow-up extended from October 1, 2007 through December 31, 2018 (maximum, 11.3 years). Age-adjusted average annual suicide mortality rates per 100 000 person-years were calculated using direct standardization. The study was approved by the Brandeis University, Colorado Multiple Institutional, Veterans Affairs Eastern Colorado Health Care System, and Vanderbilt University review boards and the Defense Health Agency Human Research Protection Program; the requirement for informed consent was waived. All analyses were run in SAS version 9.4 (SAS Institute). Statistical significance was determined at the 0.05 level. This study followed the Strengthening the Reporting of Observational Studies in Epidemiology (STROBE) reporting guideline. Additional methods are provided in Supplement 1.

## Results

VHA users (n = 488 362), compared with nonusers (n = 161 449), included higher percentages of individuals aged 40 years or older (99 538 [20.38%] vs 27 106 [16.79%]), female individuals (55 159 [11.29%] vs 15 105 [9.36%]), Black non-Hispanic individuals (84 968 [17.40%] vs 17 763 [11.00%]), and Hispanic individuals (52 659 [10.78%] vs 13 820 [8.56%]) and a lower percentage of White non-Hispanic individuals (304 608 [62.37%] vs 113 120 [70.07%]) (all *P* < .001; for more information see Table 1). There were 1850 suicide deaths (0.28%). During follow-up, age-adjusted average annual suicide rates were 28.77 (95% CI, 26.53-31.19) per 100 000 person-years among VHA users (1319 deaths [0.27%]) and 35.92 per 100 000 person-years (95% CI, 31.08-41.54) among nonusers (531 deaths [0.33%]). VHA users had a 20% lower suicide rate than nonusers (age-adjusted rate ratio, 0.80; 95% CI, 0.68-0.94; *P* = .007). Time-dependent hazard rates were higher among nonusers in the first and second years after separation, did not differ in the third year, were higher among users in the fourth year, and did not differ thereafter (Table 2).

**Table 1.** Demographic and Clinical Characteristics by Veterans Health Administration Utilization

Characteristic	VHA utilization status, No. (%)		*P* value
	Utilization (n = 488 362)	No utilization (n = 161 449)	
Demographic characteristics^a^			
Age at end of active eligibility, y			
18-24	134 350 (27.51)	50 370 (31.20)	<.001
25-29	135 625 (27.77)	47 663 (29.52)	<.001
30-34	70 195 (14.37)	22 284 (13.80)	<.001
35-39	48 654 (9.96)	14 026 (8.77)	<.001
≥40	99 538 (20.38)	27 106 (16.79)	<.001
Sex assigned in the medical record			
Male	433 203 (88.71)	146 344 (90.64)	<.001
Female	55 159 (11.29)	15 105 (9.36)	<.001
Race and ethnicity			
American Indian or Alaska Native	4763 (0.98)	1577 (0.98)	<.001
Asian American or Pacific Islander	36 439 (7.46)	13 446 (8.33)	<.001
Black non-Hispanic	84 968 (17.40)	17 763 (11.00)	<.001
Hispanic	52 659 (10.78)	13 820 (8.56)	<.001
White non-Hispanic	304 608 (62.37)	113 120 (70.07)	<.001
Other	3919 (0.80)	1270 (0.79)	<.001
Unknown	1006 (0.21)	453 (0.28)	<.001
Clinical characteristics from military medical records			
Suicide attempt	134 (0.03)	22 (0.01)	.002
Adjustment disorder	134 081 (27.46)	22 938 (14.21)	<.001
PTSD	71 959 (14.73)	7245 (4.49)	<.001
Depression or mood	100 199 (20.52)	14 878 (9.22)	<.001
Anxiety	98 008 (20.07)	13 965 (8.65)	<.001
Alcohol	55 811 (11.43)	11 761 (7.28)	<.001
Substance	29 185 (5.98)	5435 (3.37)	<.001
TBI	62 030 (12.70)	8997 (5.57)	<.001
Worst TBI recorded in military medical records			
None	426 332 (87.30)	152 452 (94.43)	<.001
Mild	17 855 (3.66)	3625 (2.25)	
Unclassified	39 337 (8.05)	4840 (3.00)	
Moderate or severe	4838 (0.99)	532 (0.33)	
Sleep disorder	169 144 (34.63)	31 189 (19.32)	<.001
Tobacco use within 1 y of end of active eligibility (n = 481 649)^b^	188 505 (51.23)	50 955 (44.81)	<.001
Chronic back pain	129 426 (26.50)	25 018 (15.50)	<.001
Chronic neck pain	41 515 (8.50)	6501 (4.03)	<.001
Chronic limb or extremity pain	203 293 (41.63)	47 955 (29.70)	<.001
Chronic fibromyalgia pain	12 264 (2.51)	1476 (0.91)	<.001
Chronic headache	56 065 (11.48)	7176 (4.44)	<.001
Chronic orofacial, ear, or temporomandibular disorder pain	2200 (0.45)	304 (0.19)	<.001
Chronic abdominal and bowel pain	34 462 (7.06)	6366 (3.94)	<.001
Chronic urogenital, pelvic, or menstrual pain	11 759 (2.41)	1888 (1.17)	<.001
Chronic musculoskeletal pain	21 429 (4.39)	3389 (2.10)	<.001
Chronic neuropathy pain	5740 (1.18)	615 (0.38)	<.001
Systemic disorders or diseases causing pain	1886 (0.39)	274 (0.17)	<.001
Other painful conditions	53 133 (10.88)	7217 (4.47)	<.001
Any of these pain conditions present	246 165 (50.41)	59 150 (36.64)	<.001

Abbreviations: PTSD, posttraumatic stress disorder; TBI, traumatic brain injury; VHA, Veterans Health Administration.

^a^ Demographic data source: Defense Manpower Data Center. Clinical characteristics are from military medical records.

^b^ Tobacco-use percentages use available group-specific records; missing data account for the reduced denominator.

**Table 2.** Time-Dependent Suicide Hazard Rates Per 100 000 Veterans^a^

Group	Years 0-1 (midpoint 0.5)	Years 1-2 (midpoint 1.5)	Years 2-3 (midpoint 2.5)	Years 3-4 (midpoint 3.5)	Years 4-5 (midpoint 4.5)	Years 5-6 (midpoint 5.5)	Years 6-7 (midpoint 6.5)	Years 7-8 (midpoint 7.5)	Years 8-11 (midpoint 9.5)
Overall	43.27 (38.21-48.33)	43.94 (38.80-49.08)	46.49 (41.08-51.89)	42.21 (36.87-47.55)	43.34 (37.64-49.04)	41.29 (35.29-47.29)	38.69 (32.26-45.12)	37.74 (30.45-45.03)	33.57 (27.51-39.62)
No VHA use	97.38 (82.15-112.62)	57.78 (45.91-69.65)	52.04 (40.42-63.67)	30.22 (20.97-39.48)	45.74 (33.65-57.83)	37.61 (25.65-49.57)	41.25 (27.18-55.33)	30.56 (16.44-44.68)	28.17 (16.12-40.21)
VHA use	25.40 (20.93-29.87)	39.42 (33.81-45.02)	44.71 (36.82-50.80)	45.96 (39.58-52.35)	42.60 (36.14-49.06)	42.39 (35.46-49.31)	37.96 (30.73-45.18)	39.72 (31.27-48.16)	35.02 (28.05-41.99)

Abbreviation: VHA, Veterans Health Administration.

^a^ Data are presented as time-dependent suicide hazard rates per 100 000 (95% CIs) estimated at the midpoint of each time window. Time is measured from the end of active Military Health System eligibility.

Among VHA users, individuals whose first VHA use occurred within 12 months had higher hazard-rate point estimates per 100 000 in years 0 to 2 than those whose first use occurred after 12 months (eg, 0-3 months: 45.48 [95% CI, 39.29-51.68]; after 12 months: 4.27 [95% CI, 1.75-6.79]). The latter estimate was based on fewer than 16 events and should be interpreted cautiously. This pattern generally persisted in years 2 to 4 and years 4 to 6; however, point estimates in the delayed-use group increased over time and were highest among the timing groups in years 6 to 11 (42.98 [95% CI, 34.60-51.36]). By utilization category, suicide hazard-rate point estimates were higher among VHA users with any VHA or VA-paid community care during the first year than among those without first-year use, with a similar pattern observed for VHA outpatient care. Individuals with first-year VHA inpatient care had substantially elevated point estimates early that remained markedly higher through later periods (eg, in years 5 to 11, with vs without first-year VHA inpatient care: 128.6 [95% CI, 84.7-172.5] vs 36.63 [95% CI, 33.04-40.23]).

## Discussion

Following military separation, age-adjusted rates of suicide differed for veterans who did and did not utilize VHA. Nearer to the time of separation, those who used VHA services had a lower suicide rate than those who did not. This is despite findings, including those presented in this letter, which suggest that those who seek VHA care are coping with more risk factors than those not seeking such care. Even among those who received VHA care, rates remained high. Interpretation is limited by selection bias, suicide misclassification, less complete clinical information for nonusers, and residual confounding by sex, race and ethnicity, baseline clinical risk, prior suicidal behavior, and indication for VHA care. Findings reflect a historical cohort, and care patterns and suicide prevention initiatives have evolved. Nonetheless, results highlighted that among those leaving Army service, risk for death by suicide, over time, differed based on use of VHA services. In the years directly posttransition, the suicide rate was lower among those who received VHA services; thereby providing support for enhanced suicide prevention care provided by the VHA.

## Notes

Supplement 1. eMethods. eReferences

Supplement 2. Data Sharing Statement
