# Supplementary material for: Veterans Health Administration Service Use and Suicide Risk After Military Separation
Source: JAMA Netw Open. 2026 Nov 5;9(11):e2642356. doi: 10.1001/jamanetworkopen.2026.42356 (PMC13741161; doi:10.1001/jamanetworkopen.2026.42356)
Supplement: Supplementary file 1 [file jamanetwopen-e2642356-s001.pdf]

Supplemental Online Content

Brenner LA, Forster JE, Hoffmire CA, et al. Veterans Health Administration service use and suicide risk after military separation. *JAMA Netw Open.* 2026;9(11):e2642356.
doi:10.1001/jamanetworkopen.2026.42356

eMethods

eReferences

This supplemental material has been provided by the authors to give readers additional information about their work.

© 2026 Brenner LA et al. *JAMA Network Open*.

eMethods

The data and follow-up period extended from October 1, 2007, through December 31, 2018; analyses were conducted from 2025 through 2026. The end of active Military Health System (MHS) eligibility was used as a proxy for separation from military service and was obtained, with demographic and military history variables, from Defense Manpower Data Center data. Race and ethnicity were self-reported in military records and limited to a single category: American Indian or Alaska Native, Asian American or Pacific Islander, Black non-Hispanic, Hispanic, White non-Hispanic, other, or unknown. A multiracial category was not available.

Approximately 76% (n = 650,072) of the SUPIC cohort separated from military service by September 2017. Because separation information was updated monthly, soldiers who died within 30 days after the recorded separation date were excluded to reduce the possibility that death occurred during military service (n = 265 [0.04%]), yielding an analytic sample of 649,811. Veterans Health Administration (VHA) use was defined by enrollment and at least 1 VHA workload encounter, inpatient stay, or encounter paid for by the Department of Veterans Affairs (VA) between separation and December 31, 2018. Individuals with utilization but no VHA enrollment were excluded. Use measures included any VHA or VA-paid community care, VHA outpatient care, and VHA inpatient care. Timing of first use was categorized as 0 to 3, 4 to 6, 7 to 9, 10 to 12, or more than 12 months after separation. Comparison of demographic, clinical and military characteristics were made using Chi-square tests.

Age-adjusted rates were standardized to the 2000 US population using age categories of 18 to 24, 25 to 29, 30 to 34, 35 to 39, and 40 years or older.1 Rates based on fewer than 16 events were considered unreliable.2 Rate ratios compared age-adjusted rates, and all estimates are reported with 95% CIs.

Time-dependent hazard rates per 100,000 persons alive at the beginning of each interval were calculated using the life-table method. Intervals began at separation and ended at suicide death or the end of follow-up. Interval lengths were selected to avoid suppression of cells with fewer than 10 events

© 2026 Brenner LA et al. *JAMA Network Open*.

and to minimize unreliable estimates when possible. All tests were 2-sided, with $P < .05$ considered statistically significant. Analyses were performed using SAS, version 9.4 (SAS Institute).

Among VHA users, time-dependent hazard rates were calculated by timing and type of VHA use during the first year after separation. Timing categories were 0 to 3, 4 to 6, 7 to 9, 10 to 12, and more than 12 months. Use types were any VHA or VA-paid community care, VHA outpatient care, and VHA inpatient care.

This study followed the Strengthening the Reporting of Observational Studies in Epidemiology (STROBE) reporting guideline for cohort studies. In addition, the study protocol was reviewed and approved by the Brandeis University Committee for Protection of Human Subjects, Colorado Multiple Institutional Review Board, VA Eastern Colorado Health Care System Institutional Review Board, Vanderbilt University Institutional Review Board, and Human Research Protection Program of the Office of the Assistant Secretary of Defense for Health Affairs/Defense Health Agency. The study was conducted in accordance with applicable federal regulations and the Declaration of Helsinki. Because the study involved secondary analysis of existing administrative data, the requirement for informed consent was waived.

© 2026 Brenner LA et al. *JAMA Network Open*.

eReferences

1 Klein, R. J. *Age adjustment using the 2000 projected US population*. (Department of Health & Human Services, Centers for Disease Control and …, 2001).

2 Division of Cancer Prevention and Control - Centers for Disease Control and Prevention. *Suppression of Rates and Counts*, <https://www.cdc.gov/cancer/uscs/technical_notes/stat_methods/suppression.htm>.

© 2026 Brenner LA et al. *JAMA Network Open*.
