# Supplementary material for: Veterans Health Administration Service Use and Suicide Risk After Military Separation
Source: JAMA Netw Open. 2026 Nov 5;9(11):e2642356. doi: 10.1001/jamanetworkopen.2026.42356 (PMC13741161; doi:10.1001/jamanetworkopen.2026.42356)
Supplement: Supplementary file 2 [file jamanetwopen-e2642356-s002.pdf]

Data Sharing Statement

Brenner. Veterans Health Administration Service Use and Suicide Risk after Military Separation. *JAMA Netw Open*. Published November 05, 2026.
doi:10.1001/jamanetworkopen.2026.42356

Data
Data available: No

Additional Information
Explanation for why data not available: Per Department of Veterans Affairs and Department of War policy.
